# Distinct Immunomodulatory Effects of Spermine Oxidase in Colitis Induced by Epithelial Injury or Infection

**DOI:** 10.3389/fimmu.2018.01242

**Published:** 2018-06-05

**Authors:** Alain P. Gobert, Nicole T. Al-Greene, Kshipra Singh, Lori A. Coburn, Johanna C. Sierra, Thomas G. Verriere, Paula B. Luis, Claus Schneider, Mohammad Asim, Margaret M. Allaman, Daniel P. Barry, John L. Cleveland, Christina E. Destefano Shields, Robert A. Casero, M. Kay Washington, M. Blanca Piazuelo, Keith T. Wilson

**Affiliations:** ^1^Division of Gastroenterology, Hepatology, and Nutrition, Department of Medicine, Vanderbilt University Medical Center, Nashville, TN, United States; ^2^Center for Mucosal Inflammation and Cancer, Vanderbilt University Medical Center, Nashville, TN, United States; ^3^Veterans Affairs Tennessee Valley Healthcare System, Nashville, TN, United States; ^4^Department of Pharmacology, Vanderbilt University Medical Center, Nashville, TN, United States; ^5^Department of Tumor Biology, Moffitt Cancer Center and Research Institute, Tampa, FL, United States; ^6^Department of Cancer Biology, The Scripps Research Institute, Jupiter, FL, United States; ^7^Johns Hopkins University, Sidney Kimmel Comprehensive Cancer Center, Baltimore, MD, United States; ^8^Department of Pathology, Microbiology, and Immunology, Vanderbilt University Medical Center, Nashville, TN, United States

**Keywords:** colitis, infection, polyamines, spermidine, spermine oxidase, mucosal immune response

## Abstract

Polyamines have been implicated in numerous biological processes, including inflammation and carcinogenesis. Homeostatic regulation leads to interconversion of the polyamines putrescine and the downstream metabolites spermidine and spermine. The enzyme spermine oxidase (SMOX), which back-converts spermine to spermidine, contributes to regulation of polyamine levels, but can also have other effects. We have implicated SMOX in gastric inflammation and carcinogenesis due to infection by the pathogen *Helicobacter pylori*. In addition, we reported that SMOX can be upregulated in humans with inflammatory bowel disease. Herein, we utilized *Smox*-deficient mice to examine the role of SMOX in two murine colitis models, *Citrobacter rodentium* infection and dextran sulfate sodium (DSS)-induced epithelial injury. In *C. rodentium*-infected wild-type (WT) mice, there were marked increases in colon weight/length and histologic injury, with mucosal hyperplasia and inflammatory cell infiltration; these changes were ameliorated in *Smox^−/−^* mice. In contrast, with DSS, *Smox^−/−^* mice exhibited substantial mortality, and increased body weight loss, colon weight/length, and histologic damage. In *C. rodentium*-infected WT mice, there were increased colonic levels of the chemokines CCL2, CCL3, CCL4, CXCL1, CXCL2, and CXCL10, and the cytokines IL-6, TNF-α, CSF3, IFN-γ, and IL-17; each were downregulated in *Smox^−/−^* mice. In DSS colitis, increased levels of IL-6, CSF3, and IL-17 were further increased in *Smox^−/−^* mice. In both models, putrescine and spermidine were increased in WT mice; in *Smox^−/−^* mice, the main effect was decreased spermidine and spermidine/spermine ratio. With *C. rodentium*, polyamine levels correlated with histologic injury, while with DSS, spermidine was inversely correlated with injury. Our studies indicate that SMOX has immunomodulatory effects in experimental colitis *via* polyamine flux. Thus, SMOX contributes to the immunopathogenesis of *C. rodentium* infection, but is protective in DSS colitis, indicating the divergent effects of spermidine.

## Introduction

Polyamines are pleiotropic and abundant aliphatic molecules found in all mammalian cells. Putrescine, spermidine, and spermine bind both RNA and DNA and are involved in various cellular processes such as maintenance of DNA structure, gene expression, RNA folding and bending, and protein translation (1–3). These three biogenic polyamines are thus essential for cell homeostasis, growth, differentiation, division, and death (4, 5). In addition, polyamines exhibit critical effects during pathological conditions through their potent toxicity at high concentration (6), regulation of inflammation (7, 8), and modulation of oxidative stress (9, 10). In this context, the homeostasis of polyamine content within a non-toxic range through synthesis, degradation, and transport is a considerable challenge for cells.

Putrescine, the first polyamine in the anabolic pathway, is generated by ornithine decarboxylation catalyzed by cytosolic ornithine decarboxylase 1 (ODC1, also known as ODC). Putrescine is then sequentially converted into spermidine and spermine *via* the action of spermidine synthase and spermine synthase, respectively (5). Importantly, the back conversion of spermine to spermidine and putrescine is a critical step in the regulation of polyamine levels, notably to limit the cytotoxic effects of spermine accumulation (11). This can be achieved by two metabolic pathways: first, the enzyme spermidine/spermine *N*(1)-acetyltransferase 1 catalyzes the acetylation of spermine into *N*(1)-acetylspermine and spermidine into *N*(1)-acetylspermidine; and then, the oxidation of these two products through the enzyme polyamine oxidase leads to the formation of spermidine and putrescine, respectively. However, this pathway is poorly expressed in the gastrointestinal tract during infection and inflammation (12–14). Second, the flavoenzyme spermine oxidase (SMOX) directly catalyzes the oxidation of spermine to spermidine and yields the generation of 3-aminopropanal and H_2_O_2_ (15). This enzyme is expressed in macrophages (9, 14, 16) and epithelial cells (9, 16, 17) during infection of the gastrointestinal tract. Moreover, it has been described that the generation of H_2_O_2_ by SMOX regulates DNA damage and apoptosis, thus supporting the development of gastric carcinoma during *Helicobacter pylori* infection (17–19) and *Bacteroides fragilis*-induced colon tumorigenesis in Min mice (16). It is important to underline that these data have been obtained by using the pharmacological SMOX inhibitor MDL 72527.

Inflammatory bowel disease (IBD) afflicts nearly two million people in the USA and is increasing worldwide (20, 21). Despite implementation of various biologic therapies, less than half of patients achieve remission. Increased understanding of the potential role of polyamines and SMOX is warranted, given the prior studies implicating SMOX in gastric inflammation and cancer (18, 19) and in human IBD (22). In this work, we questioned whether SMOX has an important role in the development of colitis. To answer this directly, we used *Smox^−/−^* mice and two models of experimental colitis: infection with *Citrobacter rodentium* and treatment with dextran sulfate sodium (DSS).

## Materials and Methods

### Animals and Models of Colitis

Experiments were conducted under protocol M/08/124 approved by the Vanderbilt University IACUC and Institutional Biosafety Committee, and the Research and Development Committee of the Veterans Affairs Tennessee Valley Healthcare System. Procedures were performed in accordance with institutional policies, AAALAC guidelines, the AVMA Guidelines on Euthanasia (CO_2_ asphyxiation followed by cervical dislocation), NIH regulations (Guide for the Care and Use of Laboratory Animals), and the United States Animal Welfare Act (1966).

C57BL/6 and C57BL/6 *Smox^−/−^*mice (23) were house-bred in our animal facility. Age-matched male wild-type (WT) and mutant mice (8–12 weeks) were used for two models of colitis: (1) mice were infected by oral gavage with 0.1 ml of LB broth containing 5 × 10^8^ *C. rodentium* DBS100 (24) as reported (25–27); feces were collected during the time course of infection and animals were euthanized after 14 days. (2) Animals were treated with 2.5% DSS (mol. wt. 36,000–50,000; TdB Consultancy) in the drinking water for 5 days; DSS was then removed, and mice were kept for 5 more days before euthanasia. In both models, mice were weighed and monitored daily and those that showed extreme distress, became moribund, or lost more than 20% of initial body weight were euthanized. After sacrifice, colons were removed, measured, cut longitudinally, cleaned, weighed, and Swiss-rolled for histology. Three proximal and distal 2-mm pieces were used for RNA and protein analysis and to determine *C. rodentium* colonization by culturing serial dilution of ground tissues on Luria–Bertani agar plates (26).

### Histological Injury Scores

Swiss-rolled colons were fixed in formalin and embedded in paraffin, and 5-µm sections were stained with hematoxylin and eosin (H&E) and examined in a blinded manner by gastrointestinal pathologists (M. Blanca Piazuelo and M. Kay Washington). For DSS colitis, inflammation severity (0–3) and inflammation extent (0–3) were each multiplied by the percent involvement (1 = 0–25%, 2 = 25–50%, 3 = 50–75%, and 4 = 75–100%) and added together to yield the inflammation score (0–24); the parameter of crypt damage (0–4) was also multiplied by the percent involvement to yield an epithelial injury score (0–16). These scores were then added together to yield the histological injury score (0–40), as described (25, 28, 29). For *C. rodentium* colitis, the histologic injury score (0–21) was the sum of acute and chronic inflammation (0–3 for each) scores multiplied by extent of inflammation (0–3) plus the epithelial injury score (0–3), as described (25, 26).

### Cytokine and Chemokine Analysis

Snap-frozen colon tissues were homogenized in CelLytic MT Cell Lysis Reagent (Sigma). Total protein concentration was determined by BCA Protein Assay (Pierce). The lysates were then analyzed using the Milliplex MAP Kit (Millipore) on a Luminex FlexMAP 3D instrument as described (Millipore) (26, 27, 30).

### Measurement of Polyamines

The concentration of the three biogenic polyamines was determined by mass spectrometry as reported (27).

### Statistics

Figures and statistics were performed using Prism 7.0a software (Graphpad Inc.). All the data represent the mean ± SEM. Data that were not normally distributed were log transformed and distribution was assessed. Student’s *t*-test or ANOVA with the Tukey test were used to determine significant differences between two groups or to analyze significant differences among multiple test groups, respectively.

## Results

### Attenuation of *C. rodentium*-Induced Colitis with *Smox* Deletion

**Figure 1:**
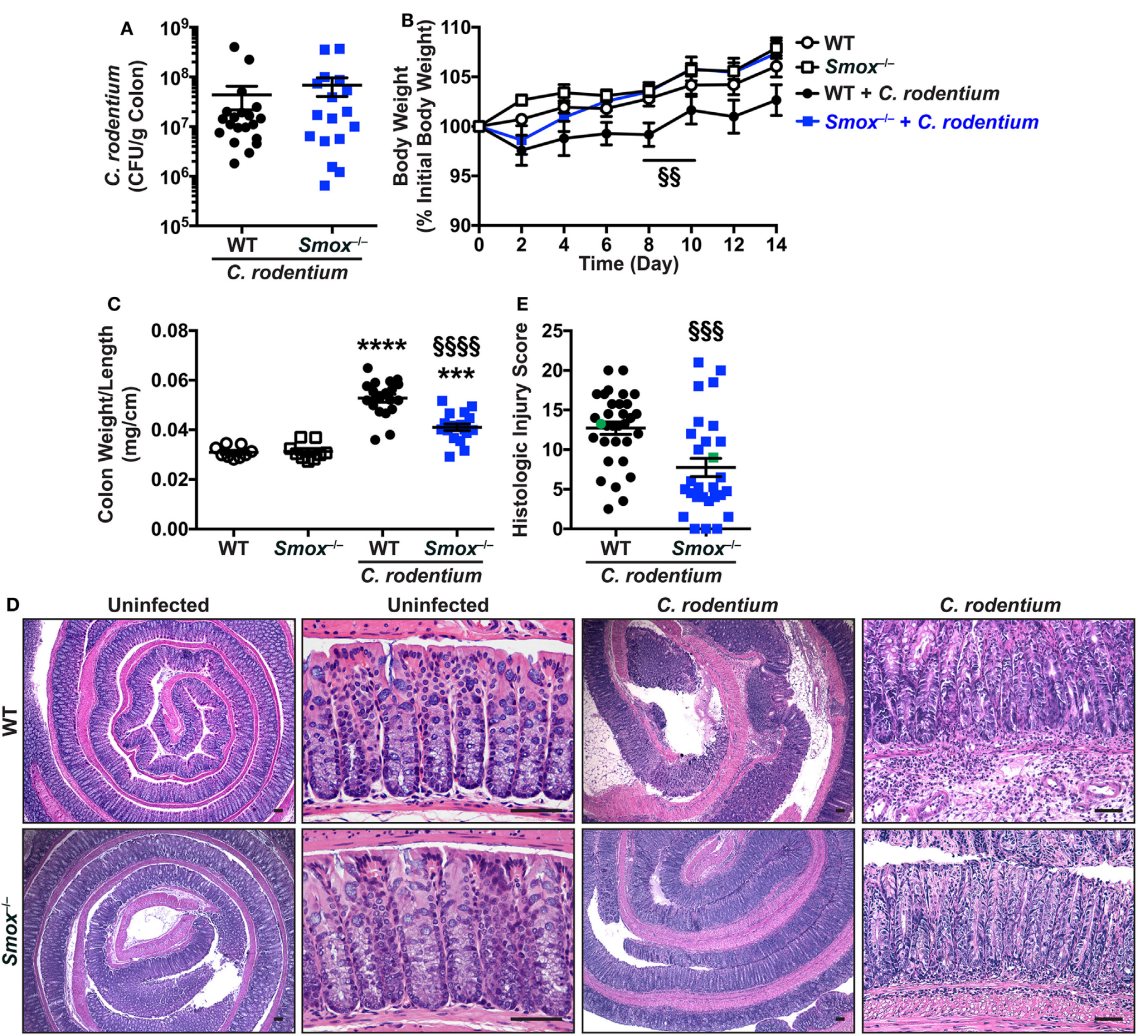
Effect of *Smox* deletion on *Citrobacter rodentium* colitis. C57BL/6 or *Smox^−/−^* mice were infected with *C*. *rodentium*. **(A)** After 14 days, *C. rodentium* colonization in the colon was assessed by plating serial dilutions. **(B)** Body weights were measured every 2 days and are presented as percentage of initial body weight. **(C)** Colons were harvested, measured, washed, and weighed. **(D,E)** Colons were Swiss-rolled and stained with hematoxylin and eosin **(D)** and scored for histologic injury **(E)**. The histologic injury score of uninfected wild-type (WT) and *Smox^−/−^* mice was 0 for both. In panel **(E)**, the green points represent the animals depicted in panel **(D)**. Scale bar, 50 µm. For panels **(B,C,E)**, ***P < 0.001 and ****P < 0.0001 compared with uninfected animals; ^§§^*P* < 0.01, ^§§§^*P* < 0.001, and ^§§§§^*P* < 0.0001 for *Smox^−/−^* mice versus WT mice infected with *C. rodentium*. Each point represents a mouse. In panel **(B)**, *N* = 8 uninfected and 15 infected mice for each genotype of animals.

We previously reported that the SMOX inhibitor MDL 72527 reduces *H. pylori*-induced gastritis and carcinogenesis (19). To now determine the role of SMOX in another infectious model of the gastrointestinal tract, we infected WT and *Smox^−/−^* mice with *C. rodentium*, the rodent equivalent of enteropathogenic *Escherichia coli* that causes colitis in mice (7, 26). *C. rodentium* colonization of the colon and levels in the feces were not affected by the deletion of *Smox* (Figure 1A; Figure S1 in Supplementary Material). When infected with *C. rodentium*, WT mice did not increase their weight to the same degree as uninfected animals (Figure 1B); by contrast, infected *Smox^−/−^* mice gained weight as uninfected mice, and there was a significant difference between the body weight of infected WT and infected *Smox-*deficient mice at day 8 and 10 post-infection (Figure 1B). Moreover, colon weight-to-length ratio, an indicator of inflammation that was increased in infected animals, was significantly attenuated by 59.3 ± 3.8% in infected *Smox^−/−^* mice compared with infected WT mice (Figure 1C). H&E staining of the colons of *C. rodentium*-infected WT mice demonstrated an effacement of the brush border, hyperplasia, severe mucosal inflammation, and submucosal edema (Figure 1D). However, the histologic damage was markedly attenuated in infected *Smox^−/−^* mice (Figure 1D). Using a comprehensive scoring system to quantify the degree of inflammation and epithelial damage (25, 26), we found a significant reduction in histologic injury in *Smox^−/−^* mice compared with WT animals (Figure 1E). There was no detectable inflammation or epithelial injury in uninfected *Smox^−/−^* or WT mice (Figure 1D).

### *Smox* Deletion Exacerbates Colitis in the DSS Model

**Figure 2:**
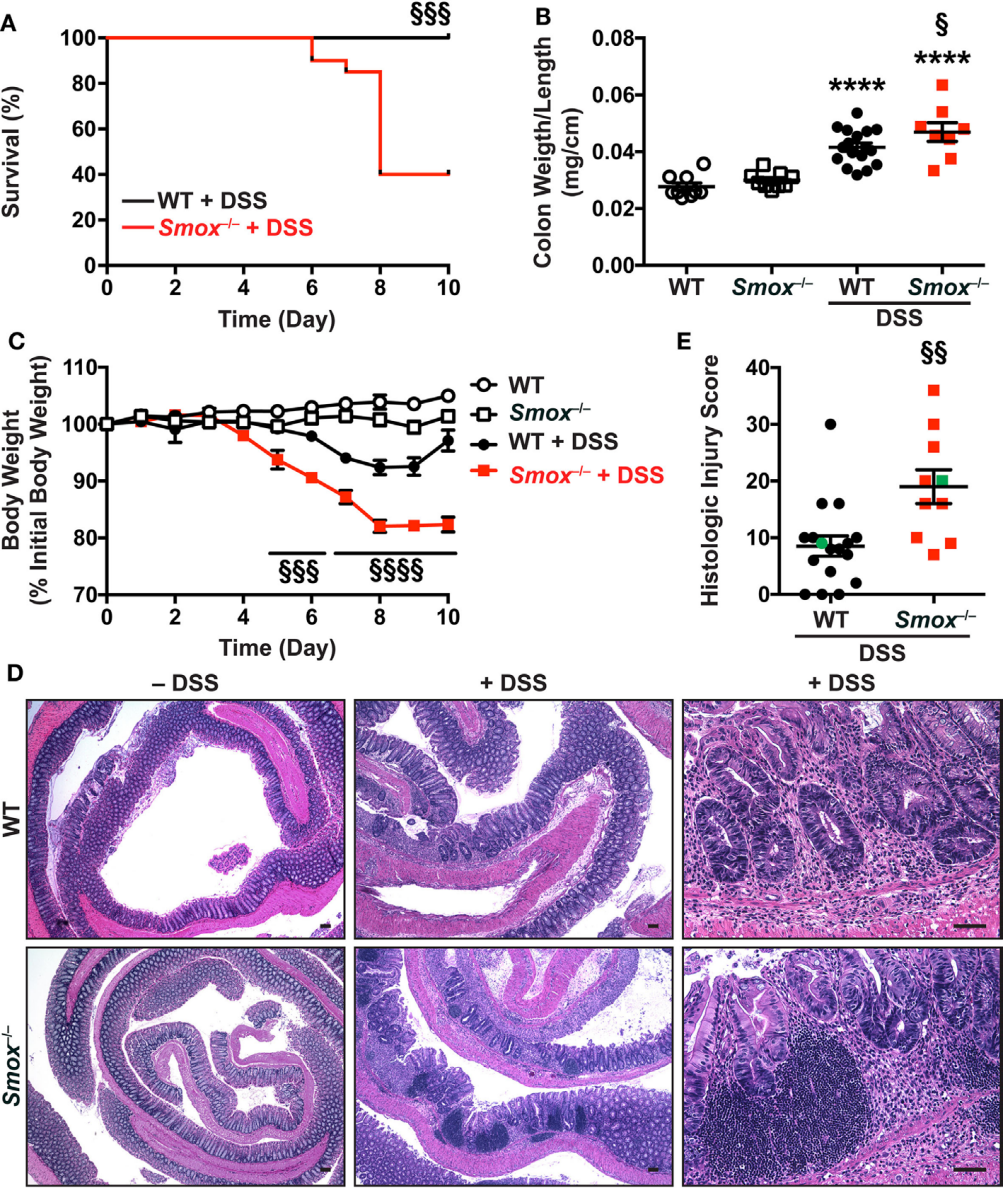
Dextran sulfate sodium (DSS) colitis in C57BL/6 mice and *Smox^−/−^* mice. Animals were treated with 2.5% DSS for 5 days and then kept for 5 more days. **(A)** Survival was monitored daily; the Kaplan–Meier plot was performed from two separate experiments [*n* = 18 wild-type (WT) and *n* = 20 *Smox^−/−^* mice]. **(B)** Colons were harvested, measured, washed, and weighed. **(C)** Body weights of all the animals in panel **(A)** were measured daily and are presented as percentage of initial body weight. **(D,E)** Colons were Swiss-rolled and stained with hematoxylin and eosin **(D)** and scored for histologic injury **(E)**. The histologic injury score of untreated WT and *Smox^−/−^* mice was 0 for both. In panel **(E)**, the green points represent the animals depicted in panel **(D)**. Scale bar, 50 µm. In all panels, ****P < 0.0001 compared with uninfected animals; ^§^*P* < 0.05, ^§§^*P* < 0.01, ^§§§^*P* < 0.001, and ^§§§§^*P* < 0.0001 versus DSS-treated WT mice.

We next treated WT and *Smox^−/−^* mice with DSS to assess the role of SMOX in a second model of experimental colitis. We used a model of injury and recovery, caused by this epithelial-damaging agent (28, 29). Surprisingly, we observed profound mortality beginning on day 6 of the 10-day protocol in the *Smox^−/−^* mice, which was not observed in DSS-treated WT animals (Figure 2A). Contrary to the *C. rodentium* model, the weight-to-length ratio of the colon was increased in *Smox^−/−^* mice compared with WT mice treated with DSS (Figure 2B). WT and *Smox^−/−^* mice began losing weight on day 6 and day 4 after starting DSS, respectively (Figure 2C). However, in accordance with the increased mortality, *Smox^−/−^* mice lost more body weight than WT mice (Figure 2C). Moreover, we observed increased overall histological injury in DSS-treated *Smox^−/−^* mice compared with WT mice with DSS colitis, with increased colonic inflammation, epithelial damage/crypt loss, and crypt abscesses (Figures 2D,E).

### The Deletion of *Smox* Affects the Mucosal Immune Response During Colitis

**Figure 3:**
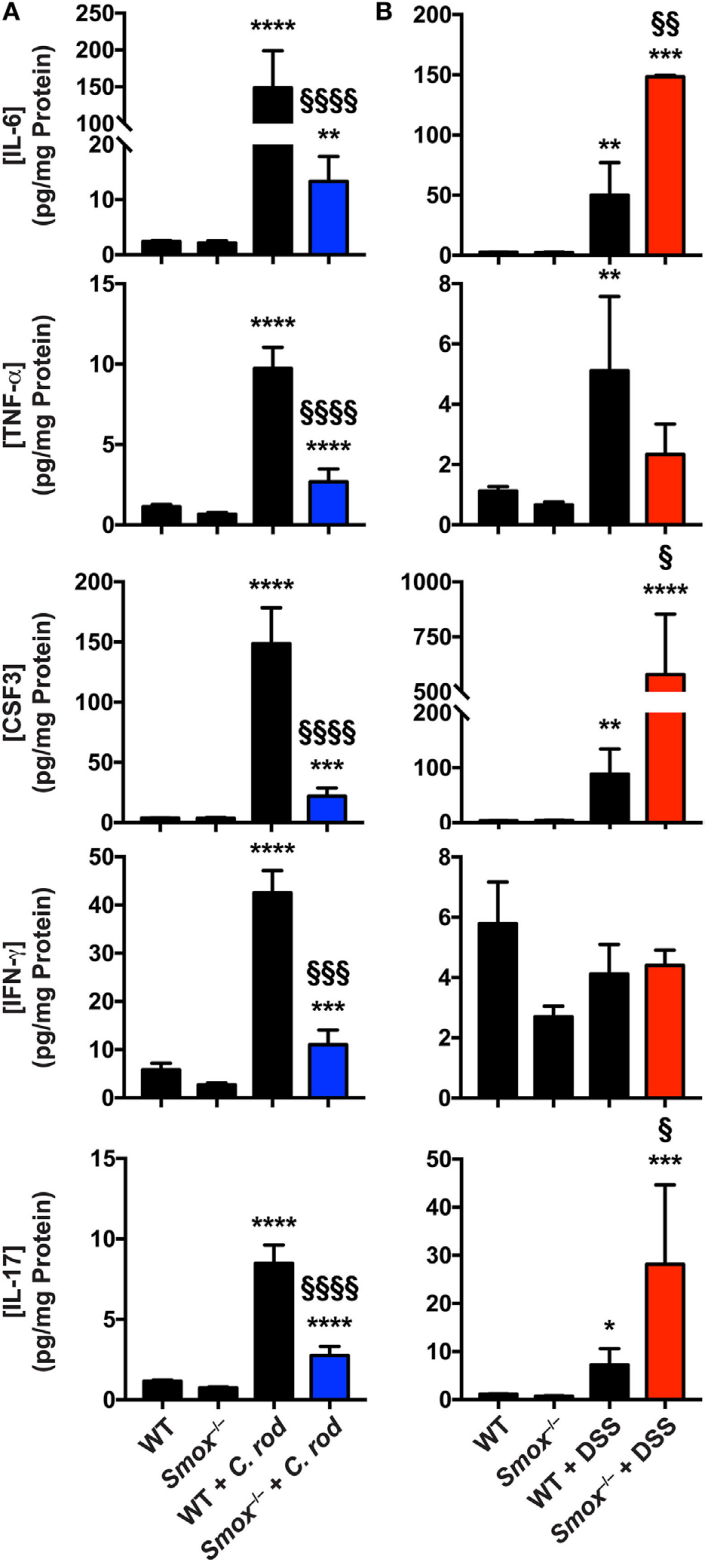
Cytokines and chemokines in colonic tissues. Protein lysates from the colons of naïve wild-type (WT) and *Smox^−/−^* mice as well as *Citrobacter rodentium*-infected mice **(A)** and dextran sulfate sodium (DSS)-treated animals **(B)** were used to determine the concentration of cytokines and chemokines by Luminex assay. *P < 0.05, **P < 0.01, ***P < 0.001, and ****P < 0.0001 compared with uninfected animals; ^§^*P* < 0.05, ^§§^*P* < 0.01, ^§§§^*P* < 0.001, and ^§§§§^*P* < 0.0001 compared with WT mice infected with *C. rodentium* infection or DSS colitis.

To further assess the role of SMOX on the immune response during colitis, we analyzed the synthesis of mediators of the inflammatory response in the colonic mucosa. As shown in Table S1 in Supplementary Material and Figure 3A, the infection of WT mice with *C. rodentium* led to an increased production of the innate cytokines IL-6, TNF-α, and CSF3 (also known as G-CSF), as well as the prototype Th1 cytokine IFN-γ, and the Th17 cytokine IL-17. Each of these cytokines was less abundant in the colons of *C. rodentium*-infected *Smox^−/−^* mice compared with infected WT animals (Figure 3A). Similarly, the chemokines CCL2, CCL3, CCL4, CXCL1, CXCL2, and CXCL10 were significantly increased in the colon of *C. rodentium*-infected WT mice compared with uninfected animals (Table S1 in Supplementary Material); these chemokines were not significantly increased in the mucosa of *Smox*-deficient mice during the infection and were decreased from the levels in the WT mice (Table S1 in Supplementary Material).

IL-6, CSF3, TNF-α, and IL-17 were also induced in the colon of DSS-treated WT mice compared to untreated animals (Figure 3B); the levels of induction were comparable to those observed in *C. rodentium* colitis (Figure 3A). However, IFN-γ was not increased in the colonic mucosa in DSS-induced colitis (Figure 3B). Notably, the effectors induced with DSS treatment, except TNF-α, were further increased in *Smox*-deficient mice that were treated with DSS (Figure 3B). The level of IFN-γ in the colon was not modulated in animals with *Smox* deletion (Figure 3B). Only the chemokines CCL2 and CXCL1 were significantly more abundant in the colon of DSS-treated animals, and there was no difference between WT and *Smox^−/−^* mice (Table S2 in Supplementary Material).

### Spermidine and Spermine Concentrations Are Regulated by SMOX in Colitis

**Figure 4:**
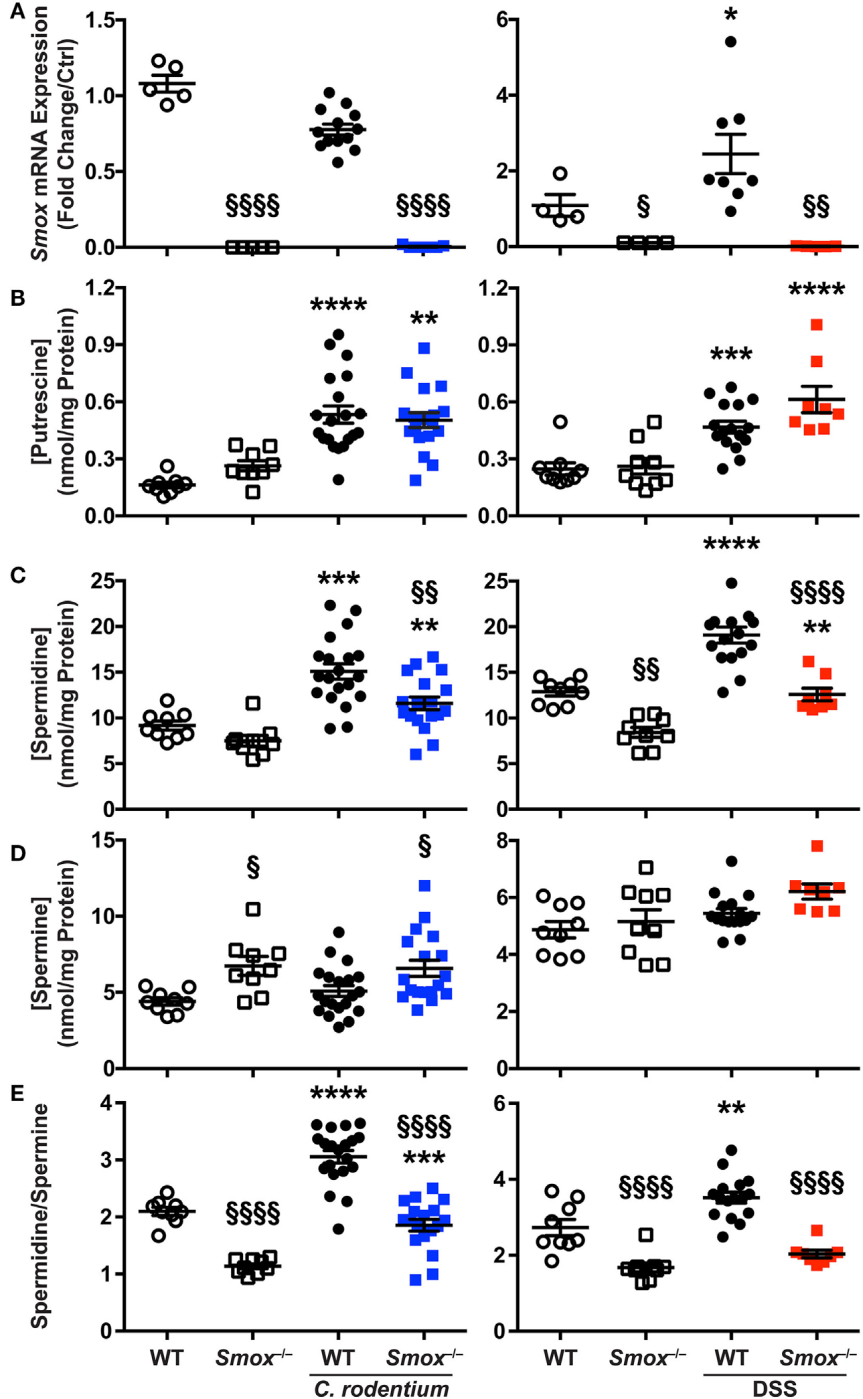
Smox expression and polyamine profiles during murine colitis. **(A)** *Smox* mRNA expression in the colon. **(B–D)** The concentration of putrescine **(B)**, spermidine **(C)**, and spermine **(D)** was determined by LC/MS in the colonic tissues. **(E)** The spermidine/spermine ratio was calculated using the values depicted in panels **(C,D)**. *P < 0.05, ***P < 0.001, and ****P < 0.0001 compared with naïve mice; ^§^*P* < 0.05, ^§§^*P* < 0.01, and ^§§§§^*P* < 0.0001 compared with wild-type (WT) mice infected with *Citrobacter rodentium* infection or treated with dextran sulfate sodium (DSS).

To further understand the role of SMOX, we analyzed the effect of *Smox* deletion on polyamine concentrations in the colon during *C. rodentium* and DSS-induced colitis. First, we found that *Smox* mRNA expression was significantly induced by 2.25 ± 0.35-fold in the colon of WT animals treated with DSS compared with untreated mice (Figure 4A); however, *Smox* upregulation was not observed during *C. rodentium* infection (Figure 4A). The deletion of *Smox* was associated with a complete elimination of *Smox* gene expression in the mouse colons in all of the naïve and colitis conditions (Figure 4A). The colonic levels of putrescine were higher in both WT and *Smox*-deficient mice infected with *C. rodentium* or treated with DSS compared with control animals, and there was no significant difference between WT and *Smox^−/−^* mice (Figure 4B). Spermidine concentration was also increased in both *C. rodentium* and DSS colitis tissues (Figure 4C). Notably, spermidine levels were significantly reduced by 31 and 54% in *Smox^−/−^* mice during *C. rodentium* infection and DSS treatment, respectively (Figure 4C). We did not observe an increase of spermine concentration in the colon of mice with either form of colitis, but there was more spermine in *Smox*-deficient mice than in WT animals in the *C. rodentium* model (Figure 4D). There was a modest increase in spermine in *Smox*-deficient mice treated with DSS, which did not reach statistical significance. Finally, the spermidine/spermine ratio was significantly increased in *C. rodentium*-infected or DSS-treated WT mice when compared with controls (Figure 4E). This ratio was significantly reduced in *Smox^−/−^* mice that were uninfected/untreated, infected with *C. rodentium*, or treated with DSS compared with WT animals (Figure 4D). These results indicate that polyamine synthesis and metabolism is enhanced during colitis and that deletion of *Smox* mainly leads to a diminution of spermidine concentration in the colon, which can likely be attributed to reduced back conversion of spermine to spermidine.

**Figure 5:**
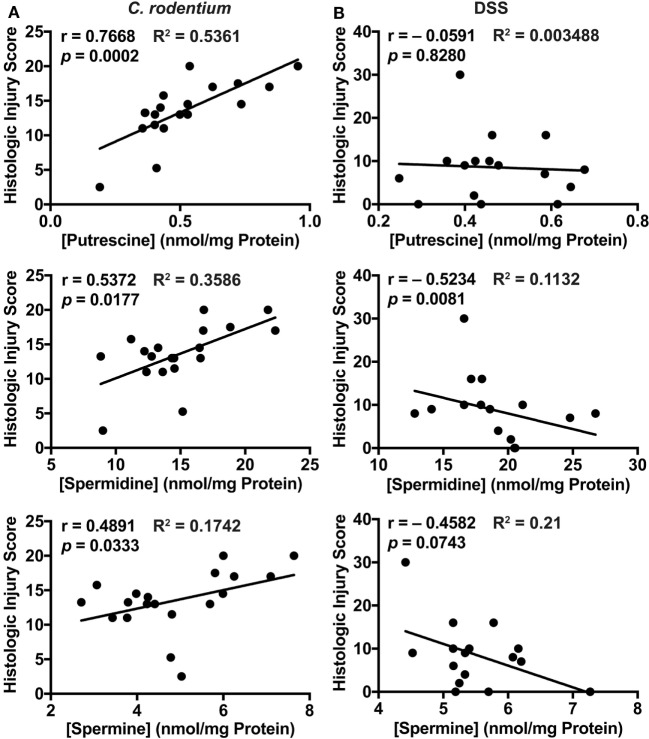
Correlation plots comparing histologic injury scores of wild-type mice (from Figures 1D and 2E) and polyamine concentrations (from Figure 4) during *Citrobacter rodentium* **(A)** or dextran sulfate sodium **(B)** colitis. Statistical analysis was performed using the Pearson correlation test.

To gain more insight into the role of polyamines in the establishment of *C. rodentium* and DSS colitis, we correlated the histopathology score with the concentration of each polyamine for each WT mouse. In the *C. rodentium* infection model, we found a positive and significant correlation between putrescine, spermidine, and spermine concentration with the injury score (Figure 5A). In contrast, we found that spermidine level in the colon was inversely correlated with histologic injury score in the DSS model (Figure 5B); thus, more spermidine is associated with decreased DSS colitis (Figure 5B). There were no significant associations between the concentration of putrescine or spermine with DSS-mediated injury (Figure 5B). These data suggest that increased polyamine synthesis is deleterious for *C. rodentium*-induced colitis, whereas spermidine protects mice during DSS-induced colitis.

## Discussion

In this study, we present compelling evidence that polyamines, and more particularly spermidine, can be considered as either beneficial or deleterious in colitis, depending on the etiology. In fact, in *C. rodentium* colitis, all three polyamines are associated with tissue damage and increased mucosal inflammation. By contrast, the generation of spermidine protects mice from DSS-induced colitis. In accordance with this result, we also found that knockout of *Smox* results in spermidine depletion and spermine accumulation in the colonic mucosa, which is associated with amelioration of *C. rodentium* colitis and worsening of DSS-induced colonic inflammation. Our data also indicate that these two models of colitis are driven by distinct pathological processes that can be potentially manipulated by the regulation of spermidine and spermine concentration.

Our laboratory has previously reported that SMOX is induced during *H. pylori* infection in macrophages (14, 31) and in gastric epithelial cells (9, 17). This leads to H_2_O_2_-mediated DNA damage (9, 17) and support of chronic inflammation, potentially due to spermine depletion (31). *C. rodentium* infection leads to the induction of ODC in the colon of mice (7, 27), which has been further underlined in this study by the increase of putrescine concentration in the colonic tissues of infected mice. However, we now observed that *Smox* is not induced in the colon of mice infected with *C. rodentium*. The discrepancy between *H. pylori* and *C. rodentium* in their ability to induce SMOX could be explained by the fact that SMOX induction in gastric tissue is dependent on the *H. pylori* virulence factor CagA (9), which is directly injected into host epithelial cells by a type IV secretion system. We can therefore postulate that *C. rodentium* does not possess the arsenal to stimulate *Smox* mRNA expression despite its numerous virulence factors including type III secretion system-dependent effectors (32). Contrasting to the *C. rodentium* model, we observed that *Smox* mRNA is upregulated in the colon of mice with DSS colitis. This is consistent with the increase of *SMOX* mRNA levels and SMOX protein expression in inflammatory cells of patients with ulcerative colitis (UC) (22). Similarly, Weiss et al. showed that spermidine was enhanced, and spermine was decreased in colonic epithelial cells of DSS-treated mice compared with untreated animals and in UC patients compared with healthy individuals (33), which is consistent with increased SMOX activity. Therefore, it would be of interest to determine which signaling pathways are involved in *Smox* mRNA expression during DSS colitis in future studies.

Spermine oxidase converts spermine into spermidine. Despite the fact that this enzyme was not upregulated in the colon of mice infected with *C. rodentium*, we found that *Smox* gene deletion resulted in a decreased concentration of spermidine and an accumulation of spermine in the colon. Similar data were obtained in the DSS model. Importantly, this effect of *Smox* deletion was also observed in uninfected/untreated animals. Altogether, these results support that the constitutive expression of SMOX, which can be augmented under specific conditions, is critical for the maintenance of spermine to spermidine flux in the colon and thus plays a role in the pathophysiology of colitis.

The next essential finding highlighted by our study is the dual role of polyamines in our two models of colitis. First, in WT mice infected with *C. rodentium*, increasing concentration of putrescine, spermidine, and spermine was significantly correlated with enhanced colonic injury score. Conversely, there was an inverse correlation between the damage to the colon and spermidine concentration in WT mice treated with DSS. These data therefore imply that polyamines are deleterious during *C. rodentium* infection and that spermidine protects animals against DSS-induced colitis. Consistently, we also observed that *Smox* deletion, which results in a reduction of spermidine concentration in the colon, is protective in *C. rodentium* colitis and exacerbates DSS-mediated injury. Although we cannot completely exclude a potential role for putrescine and spermine in the development of *C. rodentium*-induced colitis, our data support the contention that spermidine displays opposite effects in mice with intestinal infection with an attaching and effacing pathogen or with epithelial injury-driven colitis.

The question raised by our finding is how can the same polyamine have differing effects in the colon depending on the model? Supporting the concept that spermidine protects from DSS colitis, it has been reported that this polyamine exhibits anti-inflammatory properties by dampening MAPKs, PI3K, STAT-1, and NF-κB signaling pathways in myeloid cells (34–36) and thus gene transcription. Intriguingly, IFN-γ-induced synthesis of IL-6 is reduced in spermidine-treated THP-1 cells (35). This occurs through the spermidine-dependent stimulation of protein tyrosine phosphatase non-receptor type 2a that inhibits IFN-γ signaling (35). Consistent with these prior findings, it should be noted that IL-6 was one of the cytokines that we found to be further increased in *Smox^−/−^* mice during DSS colitis. Moreover, it has been described that daily supplementation of DSS-treated mice with spermidine improves clinical and histological parameters compared with sham-treated animals (35). In the same way, Yang et al. established that spermidine alleviates inflammation by favoring the polarization of macrophages toward an M2 response in a murine model of multiple sclerosis (37). The effect of spermidine on *C. rodentium* virulence gene expression is unknown and has not been analyzed in the present study. Intriguingly, it has been reported that spermidine is required for the virulence of *Salmonella enterica* Typhimurium in mice (38). Therefore, it could be now of interest whether decreased spermidine concentration in the colons of infected *Smox^−/−^* mice could lead to a reduction of *C. rodentium* virulence, and consequently to less damage without affecting colonization levels. However, the potential role of putrescine and spermine on the pathophysiology of *C. rodentium* infection should be also considered, since the concentration of all three polyamines was significantly correlated with the damage to the colon.

In conclusion, our work reveals that the local homeostasis of spermidine and spermine through SMOX is a critical regulator of colonic inflammation. In the DSS model, spermidine is protective and consequently *Smox* deletion worsens colitis. Since SMOX is induced in the colon of UC patients (22, 33), we suggest that its induction could be beneficial by increasing spermidine concentration rather than deleterious by generating H_2_O_2_. Since DSS colitis exhibits some features similar to human UC, such patients might benefit from strategies to increase spermidine concentration in the colon, rather than inhibiting SMOX. Given that spermidine supplementation is safe, may also benefit the cardiovascular system (39) and exhibits neuroprotective and anti-cancerous properties (40), the use of this polyamine as a dietary supplement should be taken into consideration for patients with UC. However, our work also underlines that spermidine supplementation most likely should be avoided in the case of gastrointestinal infections, especially those involving attaching and effacing pathogens. Since different foods contain varying amount of spermidine (41), specific dietary recommendations related to polyamine intake may help in preventing colonic inflammation and/or severe intestinal infection.

## Ethics Statement

Experimental procedures with animals were conducted under protocol M/08/124 approved by the IACUC of Vanderbilt University and the Research and Development Committee of the Veterans Affairs Tennessee Valley Healthcare System. Procedures were performed in accordance with institutional policies, AAALAC guidelines, the AVMA Guidelines on Euthanasia (CO_2_ asphyxiation followed by cervical dislocation), NIH regulations (Guide for the Care and Use of Laboratory Animals), and the United States Animal Welfare Act (1966).

## Author Contributions

NA-G, AG, and KW designed research. NA-G, KS, JS, TV, PL, CS, MA, MMA, DB, MW, and MP performed experiments. JC, CDS, and RC developed and provided *Smox^−/−^* mice. AG and KW wrote the manuscript.

## Conflict of Interest Statement

The authors declare that the research was conducted in the absence of any commercial or financial relationships that could be construed as a potential conflict of interest.
