# Supplementary material for: Distinct Immunomodulatory Effects of Spermine Oxidase in Colitis Induced by Epithelial Injury or Infection
Source: Front Immunol. 2018 Jun 5;9:1242. doi: 10.3389/fimmu.2018.01242 (PMC5996034; doi:10.3389/fimmu.2018.01242)
Supplement: Supplementary file 1 [file data_sheet_1.DOCX]

Supplementary Material

Distinct Immunomodulatory Effects of Spermine Oxidase in Colitis Induced by Epithelial Injury or Infection

**Alain P. Gobert, Nicole T. Al-Greene, Kshipra Singh, Lori A. Coburn, Johanna C Sierra, Thomas G. Verriere, Paula B. Luis, Claus Schneider, Mohammad Asim, Margaret M. Allaman, Daniel P. Barry, John L. Cleveland, Christina E. Destefano Shields, Robert A Casero, M. Kay Washington, M. Blanca Piazuelo, and Keith T. Wilson***

*** Correspondence:** [keith.wilson@vanderbilt.edu](mailto:keith.wilson@vanderbilt.edu)

**Supplementary Figure S1**

**
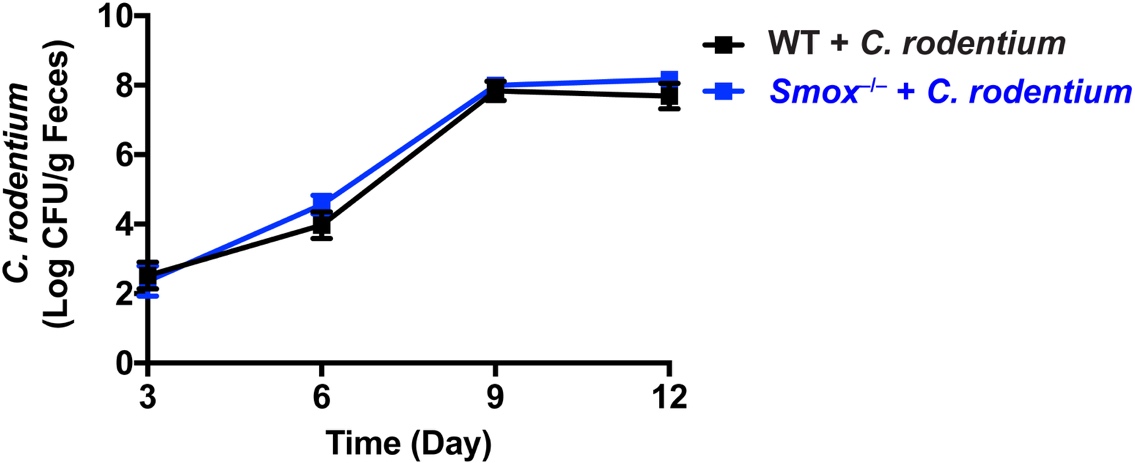
**

**Figure 1. *C*. *rodentium* burden in feces.** C57BL/6 or *Smox*^–/–^ mice were infected with *C*. *rodentium*. Feces were collected at indicated times. *C. rodentium* concentration was evaluated by plating serial dilutions of ground feces. *n* = 8 mice.

**Supplementary Table S1**

| **Analyte** | **WT** | ***Smox*^–/–^** | **WT + *C. rod*** | ***Smox*^–/–^ + *C. rod*** |
| --- | --- | --- | --- | --- |
| CCL2 | 43.28 ± 12.28 | 4.95 ± 2.57 | 117.17 ± 12.67** | 38.98 ± 10.29§§§ |
| CCL3 | 9.22 ± 0.53 | 7.56 ± 0.68 | 36.05 ± 4.10** | 15.98 ± 2.93§§ |
| CCL4 | 12.52 ± 0.89 | 7.19 ± 2.79 | 46.23 ± 4.09** | 19.59 ± 4.66§§§ |
| CCL5 | 72.49 ± 13.27 | 35.28 ± 6.09 | 56.06 ± 4.50 | 42.28 ± 4.25 |
| CXCL1 | 21.55 ± 3.09 | 19.01 ± 0.88 | 77.11 ± 5.38*** | 42.86 ± 7.47§§§ |
| CXCL2 | 11.27 ± 0.82 | 4.58 ± 0.38 | 165.17 ± 21.92** | 40.32 ± 15.66§§§ |
| CXCL10 | 34.95 ± 9.91 | 32.06 ± 5.37 | 378.07 ± 32.38** | 153.56 ± 56.82§§ |
| CSF2 | 13.73 ± 4.57 | 5.19 ± 0.41 | 13.34 ± 2.09 | 10.25 ± 2.35 |
| CSF3 | 3.37 ± 0.32 | 2.54 ± 0.57 | 148.56 ± 29.79**** | 21.90 ± 6.80***§§§§ |
| IFN-γ | 7.99 ± 1.21 | 3.18 ± 0.42 | 42.54 ± 4.60**** | 11.04 ± 3.03***§§§ |
| IL-1α | 32.43 ± 4.35 | 19.58 ± 2.88 | 54.25 ± 15.54 | 34.25 ± 6.34 |
| IL-1ß | 12.57 ± 0.07 | 5.57 ± 1.14 | 23.30 ± 3.30 | 18.66 ± 7.57 |
| IL-2 | 4.60 ± 0.25 | 3.91 ± 0.55 | 4.76 ± 0.56 | 5.11 ± 0.57 |
| IL-4 | 0.61 ± 0.12 | 0.49 ± 0.01 | 0.73 ± 0.09 | 0.67 ± 0.08 |
| IL-5 | 5.02 ± 0.61 | 5.40 ± 0.37 | 2.65 ± 0.39 | 4.09 ± 1.12 |
| IL-6 | 2.04 ± 0.22 | 1.46 ± 0.08 | 148.68 ± 50.26**** | 13.31 ± 4.51**§§§§ |
| IL-7 | 2.74 ± 0.53 | 1.48 ± 0.04 | 1.89 ± 0.41 | 2.14 ± 0.47 |
| IL-9 | 87.97 ± 29.45 | 92.22 ± 10.32 | 145.17 ± 18.54 | 143.50 ± 9.69 |
| IL-10 | 0.73 ± 0.18 | 0.53 ± 0.07 | 2.60 ± 0.50 | 2.54 ± 1.17 |
| IL-12p40 | 6.87 ± 1.34 | 2.95 ± 0.90 | 6.33 ± 2.00 | 7.02 ± 2.93 |
| IL-12p70 | 8.62 ± 1.61 | 3.67 ± 0.36 | 5.76 ± 0.85 | 4.28 ± 1.24 |
| IL-13 | 40.96 ± 1.20 | 34.72 ± 5.96 | 58.64 ± 7.69 | 65.12 ± 11.42 |
| IL-15 | 3.60 ± 0.50 | 3.02 ± 0.76 | 4.55 ± 1.52 | 5.13 ± 2.41 |
| IL-17 | 1.25 ± 0.08 | 0.65 ± 0.12 | 8.48 ± 1.15**** | 4.14 ± 1.46****§§§§ |
| TNF-α | 1.33 ± 0.20 | 0.80 ± 0.14 | 9.72 ± 1.31 | 2.68 ± 0.79 |

**Cytokine and chemokine concentrations in colon during *C. rodentium* (*C. rod*) infection.**

Values represent the mean ± SEM of the cytokine/chemokine concentrations (pg/mg total protein) in the colonic tissues, measured by Luminex assay. ***P* < 0.01, ****P* < 0.001, *****P* < 0.0001 vs*.* uninfected WT and *Smox*^–/–^ mice; §§*P* < 0.01, §§§*P* < 0.001, §§§§*P* < 0.0001 compared to WT mice infected with *C. rodentium*.

**Supplementary Table S2**

| **Analyte** | **WT** | ***Smox*^–/–^** | **WT + DSS** | ***Smox*^–/–^ + DSS** |
| --- | --- | --- | --- | --- |
| CCL2 | 6.86 ± 1.21 | 1.87 ± 0.81 | 74.35 ± 36.38*** | 49.99 ± 31.45** |
| CCL3 | 9.08 ± 0.96 | 8.36 ± 0.48 | 19.42 ± 6.03 | 14.23 ± 5.49 |
| CCL4 | 11.37 ± 1.17 | 5.63 ± 1.72* | 19.74 ± 7.05 | 16.59 ± 8.32 |
| CCL5 | 40.72 ± 6.02 | 16.21 ± 0.90 | 35.42 ± 8.90 | 21.02 ± 9.23 |
| CXCL1 | 23.25 ± 3.92 | 24.49 ± 0.57 | 75.40 ± 36.36*** | 73.96 ± 33.19*** |
| CXCL2 | 8.69 ± 2.69 | 5.84 ± 0.15 | 118.26 ± 54.24 | 70.40 ± 33.66 |
| CXCL10 | 28.28 ± 2.31 | 20.33 ± 3.65 | 40.92 ± 9.27 | 35.77 ± 13.89 |
| CSF2 | 7.40 ± 0.81 | 5.80 ± 0.34 | 18.79 ± 9.72 | 6.20 ± 1.46 |
| CSF3 | 3.58 ± 0.21 | 3.57 ± 0.61 | 88.43 ± 45.72** | 578.75 ± 274.90****§ |
| IFN-γ | 5.79 ± 1.38 | 2.70 ± 0.35 | 8.50 ± 4.45 | 4.41 ± 0.50 |
| IL-1α | 26.31 ± 3.97 | 19.02 ± 2.08 | 47.21 ± 15.53 | 35.48 ± 6.13 |
| IL-1ß | 5.57 ± 0.20 | 3.78 ± 0.70 | 31.39 ± 17.02 | 12.83 ± 4.24 |
| IL-2 | 5.83 ± 0.49 | 6.07 ± 0.31 | 5.38 ± 1.30 | 4.34 ± 0.83 |
| IL-4 | 0.93 ± 0.16 | 0.68 ± 0.03 | 1.43 ± 0.58 | 0.73 ± 0.15 |
| IL-5 | 6.05 ± 0.52 | 7.63 ± 0.46 | 4.19 ± 0.94 | 21.12 ± 0.20**§§ |
| IL-6 | 2.37 ± 0.21 | 2.15 ± 0.38 | 50.11 ± 26.93** | 100.97 ± 47.43***§§ |
| IL-7 | 1.66 ± 0.13 | 1.52 ± 0.36 | 4.67 ± 2.89 | 2.18 ± 0.52 |
| IL-9 | 145.04 ± 5.06 | 185.11 ± 5.26 | 151.95 ± 12.78 | 182.16 ± 28.82 |
| IL-10 | 1.01 ± 0.38 | 1.22 ± 0.28 | 2.65 ± 0.64* | 4.26 ± 2.19**§ |
| IL-12p40 | 10.11 ± 7.31 | 5.18 ± 1.59 | 22.59 ± 14.85 | 8.76 ± 2.74 |
| IL-12p70 | 9.76 ± 3.06 | 4.08 ± 0.91 | 8.19 ± 2.95 | 4.18 ± 1.95 |
| IL-13 | 95.67 ± 14.31 | 98.14 ± 17.93 | 89.03 ± 20.35 | 36.01 ± 11.33 |
| IL-15 | 19.07 ± 0.23 | 5.10 ± 2.27 | 16.67 ± 11.48 | 2.27 ± 1.06 |
| IL-17 | 1.16 ± 0.07 | 0.74 ± 0.07 | 7.24 ± 3.35* | 24.83 ± 18.34***§ |
| TNF-α | 1.13 ± 0.14 | 0.66 ± 0.09 | 5.11 ± 2.46** | 2.33 ± 1.01 |

**Cytokine and chemokine concentrations in colonic tissues in DSS colitis**

Values represent the mean ± SEM of the cytokine/chemokine concentrations (pg/mg total protein) in the colonic tissues, measured by Luminex assay. **P* < 0.05, ***P* < 0.01, ****P* < 0.001, *****P* < 0.0001 vs*.* control mice; §*P* < 0.05, §§*P* < 0.01 vs. WT mice treated with DSS.
